# Absolute and relative preferences for mobile phone internet content, mobile phone dependence, and depressive symptoms: a study of Chinese university students in the post-pandemic era

**DOI:** 10.3389/fpubh.2023.1247438

**Published:** 2023-10-12

**Authors:** Hua Yang, Zhikang Wang, Yujie Jiang, Jie Tang

**Affiliations:** ^1^School of Marxism, Chongqing University, Chongqing, China; ^2^School of Public Policy and Administration, Chongqing University, Chongqing, China

**Keywords:** absolute preference, mobile phone internet content, mobile phone dependence, relative preference, depressive symptoms, Chinese university students

## Abstract

**Background:**

Due to the COVID-19 pandemic, Chinese university students may have increased mobile phone dependence, a habitual behavior in the student population and a risk factor for depressive symptoms. Therefore, this study explored the association between mobile phone dependence and depressive symptoms in Chinese university students in the post-pandemic era. It also investigated the effects of different types and categories of mobile phone Internet content preferences. In particular, this study examined whether mobile phone dependence mediates the relationship between absolute preference (AP) for mobile phone Internet content and depressive symptoms, and whether relative preference (RP) for mobile phone Internet content moderates the association between mobile phone dependence and depressive symptoms.

**Methods:**

A cross-sectional study of Chinese university students recruited through Credamo was conducted in February–March, 2023. Participants completed the Mobile Phone Internet Content Preference Questionnaire, Self-rating Questionnaire for Adolescent Problematic Mobile Phone Use, and Center for Epidemiological Survey, Depression Scale. Statistical analyses included descriptive statistics, correlation, regression, and analyses of mediation and moderation effects. The final sample comprised 1,602 students (709 males).

**Results:**

We found a positive association between mobile phone dependence and depressive symptoms. The mediating role of mobile phone dependence between AP for mobile phone Internet content and depressive symptoms differed according to the type and category of content. Meanwhile, different types and categories of RP for mobile phone Internet content moderated the association between mobile phone dependence and depressive symptoms in opposite directions.

**Conclusion:**

Our results highlight the interrelationships among mobile phone Internet content preferences, mobile phone dependence, and depressive symptoms in Chinese university students. For different types and categories of mobile phone Internet content preferences, we propose distinct preventive measures to alleviate students’ depressive symptoms.

## 1. Introduction

Levels of depression, a common psychological disorder among university students, have remained high in recent years. According to survey data from the 2022 Blue Book on National Depression, nearly 50% of China’s 95 million depressed patients are university students (1). As a serious mental illness with high prevalence and recurrence rates (2), long-term depression may result in life-threatening behaviors, such as alcoholism, self-injury, and even suicide and homicide (3). Since the onset of the COVID-19 pandemic in 2020, mobile phone use by Chinese university students has increased, bringing major changes to their study, work, and life. Online learning and home quarantine have reduced interpersonal communication and interaction among students and their teachers, classmates, relatives, and friends. Meanwhile, poor learning efficiency, low grades, and mobile phone overuse can lead to mobile phone dependence and negative emotions, such as fatigue and guilt, thus causing depression among university students. In the post-pandemic era, students continue to use mobile phones excessively. However, the association between mobile phone dependence and depressive symptoms in different studies is contradictory, being reported as inhibited (4), unrelated (4, 5), or facilitated (6), which may be related to the research context and population. Thus it is necessary to explore the association between mobile phone dependence and depressive symptoms among Chinese university students in the post-pandemic era. Busch (7) proposed that various moderators may explain these conflicting findings; however, little is known about these moderators in previous research. In addition, scholars have focused on different categories and types of mobile phone Internet content and motivation to deepen the understanding of mobile phone dependence (4, 8). Therefore, this study aimed to examine the mediating role of mobile phone dependence between absolute preferences (APs) for mobile phone Internet content and depressive symptoms, and the moderating role of relative preferences (RPs) for mobile phone Internet content between mobile phone dependence and depressive symptoms among Chinese university students in the post-pandemic era. Empirical evidence on this topic can guide efforts to relieve negative emotions and maintain psychological balance in Chinese university students.

### 1.1. Mobile phone dependence and depressive symptoms among Chinese university students

Compared with Europe, the United States, Japan, and South Korea, China is characterized by a higher preference for accessing the Internet via mobile phone. At year-end 2022, the number of Chinese Internet users had reached 1.067 billion, of which 99.8% used mobile phones to access the Internet—65.7% higher than the proportion accessing internet via a computer (9). Since the onset of the pandemic in 2020, online learning and home quarantine have prompted Chinese university students to study, work, and live online through mobile phones. Despite the benefits of information access, convenient communication, and use of sports Apps to promote exercise behaviors and weight control for health (10), possible consequences of excessive mobile phone use include mobile phone dependence and multiple physiopathological symptoms, such as headache, fatigue, and poor concentration (11), as well as impaired psychological functions, such as anxiety and depression (12). Therefore, in the post-pandemic era, mobile phone dependence may trigger negative emotions and lead to depression among Chinese university students. In particular, mobile phone dependence constitutes inappropriate use of mobile phones and is considered as technology addiction or a behavior that can lead to serious social and emotional disorders (13). Meanwhile, for the purpose of this article, depressive symptoms refer to symptoms associated with depression, a sub-healthy state in the general population, rather than a clinical diagnosis of mental illness (14).

Multiple studies have explored the association between mobile phone dependence and depressive symptoms among university students. For example, a positive association between mobile phone dependence and depressive symptoms has been reported for adolescents and adults in Hong Kong and Taiwan, China (15, 16). However, studies with participants from Japan (5) and South Korea (4) have found either no or even a negative association between the duration of mobile phone use and depressive symptoms in adolescents. Evidently, the association between mobile phone dependence and depressive symptoms may vary in different research contexts and populations. Studies of university students in the Chinese mainland suggest that mobile phone dependence becomes compulsive during the process of reinforcement of mobile phone use, which could cause individuals to start experiencing negative emotions when not engaging in the behavior. These findings indicate a positive association between mobile phone dependence and depressive symptoms among Chinese university students (6, 17). Another longitudinal study found a bidirectional and positive association between mobile phone dependence and depressive symptoms, but only in the female population (18). However, these studies focused on regional samples and did not include senior university students, so there remains a lack of comprehensive, nationwide evidence on the association. A meta-analysis (*n* = 33,650) with Chinese university students as the main study population showed that university students with mobile phone dependence had worse sleep quality and were more likely to exhibit high levels of anxiety, depression, and impulsivity, seemingly providing conclusive evidence for a positive association between mobile phone dependence and depressive symptoms among Chinese university students (19). Based on the above analysis, we propose the first hypothesis:

*H1:* Mobile phone dependence is positively associated with depressive symptoms among Chinese university students in the post-pandemic era.

### 1.2. Absolute preference for mobile phone internet content, mobile phone dependence, and depressive symptoms

AP for Internet content refers to the extent to which audience access a particular type of information online (20). Different types of AP for mobile phone Internet content among Chinese university students are likely to influence their mobile phone dependence and even depressive symptoms. According to Zhao’s (21) classification system of mobile phone Internet contents, students’ preferences are divided into six information categories: entertainment (e.g., short videos, games, music, and sports); lifestyle (e.g., shopping, food, and travel), learning (e.g., essays, course knowledge, foreign languages, and software); work (e.g., internships, recruitment, and part-time jobs); political news (e.g., national and international politics); and social (e.g., environmental protection, rich–poor gap, and medical care). These six categories are also organized into three information types: entertainment and lifestyle are the stress-reducing type; learning and work are the stress-producing type; and political news and social are the neutral type (meaning that reading such information does not directly reduce or produce stress). Previous studies have shown that the different types and categories of Internet content preferences have different effects on Internet addiction (22, 23). Accordingly, the present study explored the associations of different types and categories of phone Internet contents with mobile phone dependence and depressive symptoms among Chinese university students.

Numerous studies have explored the key factors and mechanisms of the formation of mobile phone dependence in university students, considering demographic characteristics, such as age, education, and gender (24, 25); personality characteristics, such as openness and impulsiveness (26, 27); individual factors, such as communication needs and entertainment needs (28, 29); and environmental factors, such as interpersonal relationships, family economic level, mobile phone use in peer groups, and social interaction (30, 31). However, previous studies have focused on the direct effects of individual and environmental factors, while neglecting the interactions between them. In particular, previous studies have not evaluated how individuals’ AP of mobile phone Internet contents affects mobile phone dependence and, in turn, depressive symptoms. Studies have found that people are prone to flow experiences when individual abilities are matched with task challenges (32), and AP for different types and categories of mobile phone Internet contents, such as entertainment and news, can produce flow experiences (33). According to the flow theory, flow experiences can produce positive outcomes, such as full attention on the current activity, action and perceptual fusion, familiarity, and enjoyment. Consequently, individuals in a flow state perceive that time flies (34) and benefit from improved satisfaction and subjective well-being (35), as well as reduced depressive symptoms. Accordingly, we propose the following hypotheses:

*H2a:* AP for the stress-reducing type of mobile phone Internet contents (AP-SRTm) significantly negatively predicts depressive symptoms among Chinese university students in the post-pandemic era.

*H2b:* AP for the stress-producing type of mobile phone Internet contents (AP-SPTm) significantly negatively predicts depressive symptoms among Chinese university students in the post-pandemic era.

*H2c:* AP for the neutral type of mobile phone Internet contents (AP-NTm) significantly negatively predicts depressive symptoms among Chinese university students in the post-pandemic era.

However, some studies suggest that flow experiences may lead to negative outcomes, such as overuse or even addiction. For example, people are easily attracted to and immersed in stress-reducing mobile phone Internet contents, such as short videos and online games, which can lead to mobile phone dependence (36). Prolonged use may produce feelings of exhaustion and loss (37), and some Internet users experience guilt or even depressive symptoms after flow experiences (38). Some studies have found that Internet content categories such as entertainment are more likely to cause Internet addiction and dependence in adolescents (22). Based on this, we propose the following hypothesis:

*H3a:* AP-SRTm significantly positively predicts mobile phone dependence, which suppresses the positive predictive effect of AP-SRTm on depressive symptoms among Chinese university students.

Meanwhile, studies have shown that university students are easily distracted by stress-reducing mobile phone Internet contents when accessing and processing stress-producing mobile phone Internet contents, such as learning and work information, leading to mood swings, reduced interest in learning, and other negative outcomes (39). This indicates that university students prefer the stress-reducing type to the stress-producing type of mobile phone Internet contents; while the former is more likely to cause mobile phone dependence, the latter may be negatively associated with mobile phone dependence. Students access learning and work information with the aim of solving real-life problems in study and work. As such information brings limited pleasure, students may cease accessing it after solving these problems. Therefore, we propose the following hypothesis:

*H3b:* AP-SPTm significantly negatively predicts mobile phone dependence, which plays a mediating role between AP-SRTm and depressive symptoms among Chinese university students.

AP-NTm refers to university students’ use of various search engines to find and gain insights into political news and social issues. Some studies of adolescent groups have shown that these information categories are similar to the stress-producing type, and are accessed for real-life purposes (22). As neutral-type information can bring only limited pleasure to adolescents, it is unlikely to lead to mobile phone dependence. However, some scholars argue that university students have a stronger demand to acquire this type of information compared to adolescents, given their deeper interest in and understanding of politics and society (23). The neutral type of mobile phone Internet content can also bring pleasure and satisfaction to university students, driving them to invest more time and energy, potentially leading to mobile phone dependence. Recognizing the intersecting age groups of university students and adolescents, we propose the following competing hypotheses:

*H3c:* AP-NTm significantly positively predicts mobile phone dependence, which plays a mediating role between AP-NTm and depressive symptoms among Chinese university students.

*H3d:* AP-NTm does not significantly predict mobile phone dependence, which, therefore, does not play a mediating role between AP-NTm and depressive symptoms among Chinese university students.

### 1.3. Mobile phone dependence, relative preference for mobile phone internet content, and depressive symptoms

RP for Internet content refers to the extent to which an individual prefers accessing a particular type of information online rather than through offline channels (40). It has the same three types and six categories as for AP for Internet content. The association between mobile phone dependence and depressive symptoms in university students may vary depending on their RP for mobile phone Internet content. Many studies have explored moderators of the direct and indirect pathways between mobile phone dependence and depressive symptoms, but some moderators (e.g., gender) do not appear to have a statistically significant effect, so it is important to explore other moderating variables (17).

According to the third-person effect theory, when people access information eliciting negative emotions, they overestimate its influence on others while underestimating its influence on themselves, perceiving some level of personal immunity to negative information (41). This serves to optimize an individual’s emotional state. Conversely, for information eliciting positive emotions, people do not perceive a significant difference between the effect on themselves and on others (42), or may even overestimate its personal influence while underestimating its influence on third persons (43). Meanwhile, some studies also suggest that the intensity of the third-person effect is not absolute and can be influenced by factors such as the personal characteristics of the audience (44) and the source of information (45). In addition, the level of social distance significantly affects the intensity of the third-person effect of Internet information (46), with intensity increasing as the social distance increases between the audience and others (47). Some scholars point out that social distance is both vertical, in terms of economic and political status, and horizontal, in terms of the closeness of interactions (48). The degree to which an individual exchanges a certain type of information offline with others in daily life can reduce the horizontal social distance between the audience and others, thus reducing the third-person effect of mobile phone Internet information and regulating the individual’s psychological state.

Another key consideration is that RP for different types and categories of mobile phone Internet content, such as entertainment, learning, and work information, has different effects on negative and positive emotions. University students with mobile phone dependence prefer stress-reducing mobile phone Internet content, such as entertainment and lifestyle information, because it tends to elicit positive emotions (49). In the short term, exposure to stress-reducing mobile phone Internet content can produce flow experiences and positive emotions. However, in the long term, accessing stress-reducing mobile phone Internet content (i.e., entertainment and lifestyle information) can easily trigger a sense of time distortion and a series of negative emotions, such as fatigue, emptiness, and psychological guilt. According to the third-person effect theory, short-term access to stress-reducing mobile phone Internet content can elicit positive emotions, whereas individuals may even overestimate the long-term effect, thus overestimating the long-term negative effect of stress-reducing mobile phone Internet content. Higher RP for the stress-reducing type of mobile phone Internet contents (RP-SRTm) will increase social distance and intensify the third-person effect, which will increase negative emotions in the long term. Therefore, we propose the following hypothesis:

*H4a:* RP-SRTm positively moderates the association between mobile phone dependence and depressive symptoms among Chinese university students.

According to the third-person effect theory, RP of mobile phone Internet content increases social distance and enhances the third-person effect, while mobile phone dependence and both the stress-producing type and neutral type of mobile phone Internet content may bring negative information and produce negative emotions in university students, thus affecting their psychological health (50). On this basis, university students may perceive themselves as immune to the negative influence, thus increasing positive emotions and reducing depressive symptoms. Therefore, we propose the following hypotheses:

*H4b:* RP-SPTm negatively moderates the association between mobile phone dependence and depressive symptoms among Chinese university students.

*H4c:* RP-NTm negatively moderates the association between mobile phone dependence and depressive symptoms among Chinese university students.

The main purpose of this study was to investigate the interrelationships among mobile phone dependence, depressive symptoms, and AP and RP for different types and categories of mobile phone Internet content in a sample of Chinese university students. Our findings will provide new theoretical and empirical bases for efforts to relieve negative emotions and maintain psychological balance in the student population in the post-pandemic era.

## 2. Materials and methods

### 2.1. Study objectives

Between February and March, 2023, we used the Credamo survey platform to distribute questionnaires to 60 university students in each of the 30 provinces of China. The initial sample included 1,800 Chinese university students. All students have signed informed consent online. We explained the purpose, procedure, and privacy of this study on the homepage of the questionnaire. Participants were told that our study does not present any potential risks and discomforts, and they can withdraw from our study at any time. Participants must check the “I have read the information provided and am willing to participate in this study” option before participating in the survey. To ensure that only university students completed the questionnaire, we asked Credamo to send out online invitations only to university students in its subject pool. We discarded questionnaires with many missing answers or identical responses. In addition, we also added reverse worded items to prevent participants from acquiescent answering, and discarded questionnaires with inconsistent answers. After excluding invalid responses, the final sample size was 1,602, representing a valid response rate of 89.0%.

### 2.2. Measures

#### 2.2.1. Absolute preference and relative preference for mobile phone internet content

Based on Prior’s (51) method, we determined the AP for mobile phone Internet content based on the frequency of university students’ exposure to certain types and categories of information through mobile phones. We devised The Mobile Phone Internet Content Preference Questionnaire that comprises 12 items. Six items evaluate the frequency of exposure to each type/category of information through mobile phones, which represents a specific type/category of AP for mobile phone Internet content. The remaining six items evaluate the frequency of offline communication with others about each type/category of information. We determined the RP for mobile phone Internet content based on the difference between the frequency of students’ exposure to a certain type/category of information through mobile phones and the frequency of their offline communication with others about the same type/category of information (52). Participants responded to each item on a 5-point Likert scale: 1 = “Never,” 2 = “Occasionally,” 3 = “Sometimes,” 4 = “Often,” and 5 = “Every day.” A score of 3 or more indicates that the student has an AP for accessing this type/category of information through mobile phones; a score of 1 or 2 indicates that the student has a RP for accessing this type/category of information through mobile phones, rather than offline communication. The Cronbach’s alpha coefficient was 0.722 for the AP for mobile phone Internet content scale and 0.701 for the RP for mobile phone Internet content scale.

#### 2.2.2. Mobile phone dependence

We evaluated mobile phone dependence using the Self-rating Questionnaire for Adolescent Problematic Mobile Phone Use, as devised by Tao et al. (53). The instrument comprises 13 questions, each answered on a 5-point Likert scale: 1 = “Not at all,” 2 = “Not quite,” 3 = “Not sure,” 4 = “Quite consistent,” and 5 = “Very consistent.” A total score of 28 or more indicates the existence of mobile phone dependence, while higher scores indicate a higher level of mobile phone dependence. The Cronbach’s alpha coefficient was 0.880.

#### 2.2.3. Depressive symptoms

Depressive symptoms were evaluated using the Center for Epidemiological Survey, Depression Scale, originally developed by Radloff (14) and adapted for a Chinese population by Zhang et al. (54). The scale comprises 20 questions, of which 16 are related to negative emotions and 4 are reverse-scored items related to positive emotions. For each item, participants were asked to report the extent to which they had felt as described in the week before the survey: “Not or almost not” for less than 1 day (score: 0); “Rarely” for 1–2 days (score: 1); “Often” for 3–4 days (score: 2); and “Always” for 5–7 days (score: 3). We interpreted total scores of ≤ 15 as indicating no depressive symptoms, 16–19 as possible depressive symptoms, and ≥ 20 as definite depressive symptoms. The Cronbach’s alpha coefficient was 0.913. All the research instruments above were validated for Chinese language.

### 2.3. Data processing and analysis

SPSS 24.0 was used to perform the common method bias test and descriptive statistics. We tested *H1* using Pearson correlations. To test the proposed mediating effects, we used model 4 of the Hayes PROCESS macro 3.5 for SPSS (55), setting the number of Bootstrap iterations to 5,000. Finally, model 1 of the PROCESS macro 3.5 was used to test the moderating effects. In both the mediation and moderation tests, we controlled for the demographic variables, including gender, grade, and major.

## 3. Results

### 3.1. Common method bias test

As all the data were obtained from self-report measures, they may be affected by common method bias. The results of Harman’s single-factor test identified eight factors with original root >1. The first factor explained 22.971% of the total variance, which is below the critical threshold of 40%. This indicates that the common method bias did not significantly affect our results.

### 3.2. Descriptive statistics

Of the final sample of 1,602 Chinese university students, 709 (44.3%) were males and 893 (55.7%) were females. Regarding participants’ major, 787 (49.1%) were students of liberal arts (e.g., literature, history, economics, and law), 363 (22.6%) were students of science (e.g., mathematics, physics, chemistry, and biology), and 452 (28.2%) were students of engineering (e.g., mechanical engineering, electrical engineering, architecture, and communication). With regard to the grade, 205 (12.8%) participants were freshmen, 390 (24.3%) were sophomores, 466 (29.1%) were juniors, 301 (18.8%) were seniors, 213 (13.3%) were graduate students, and 27 (1.7%) were doctoral students.

Participants’ AP for mobile phone Internet content was highest for entertainment information (97%) and lifestyle information (96%), both of which reduce stress, followed by the stress-producing type learning information (93%). The RP for mobile phone Internet content was highest for the neutral type political news information (69%) and the stress-producing type learning information (62%). Mobile phone dependence was found in 61% of the students. Furthermore, 15% of participants were likely to have depressive symptoms and 34% definitely had depressive symptoms (see Table 1).

**Table 1:** Descriptive statistics of mobile phone internet content preferences, mobile phone dependence, and depressive symptoms among Chinese university students with different demographic characteristics (*n* = 1,602).

Scale	Number	Percentage	AP (≥ 3)												RP (≥ 1)												MPD (≥ 28)		DS			
			AP-SPTm				AP-NTm				AP-SRTm				RP-SPTm				RP-NTm				RP-SRTm						Definite (≥ 20)		Likely (16–19)	
			Learning		Work		Social		Political news		Entertainment		Lifestyle		Learning		Work		Social		Political news		Entertainment		Lifestyle							
	*N*	%	*N*	%	*N*	%	*N*	%	*N*	%	*N*	%	*N*	%	*N*	%	*N*	%	*N*	%	*N*	%	*N*	%	*N*	%	*N*	%	*N*	%	*N*	%
	1,602	100	1,482	93	1,272	79	1,276	80	1,232	78	1,554	97	1,536	96	994	62	730	46	917	57	1,112	69	887	55	784	49	970	61	538	34	245	15
Grade																																
Freshman	205	13	182	89	129	63	161	79	148	72	193	94	194	95	124	60	105	51	127	62	134	65	112	55	90	44	129	63	74	36	43	21
Sophomore	390	24	362	93	297	76	317	81	306	78	380	97	374	96	244	63	201	52	221	57	282	72	210	54	209	54	218	56	133	34	50	13
Junior	466	29	428	92	374	80	373	80	359	77	453	97	449	96	293	63	200	43	255	55	317	68	259	56	221	47	283	61	155	33	74	16
Senior	301	19	282	94	268	89	239	79	238	79	294	98	291	97	191	63	120	40	177	59	214	71	168	56	149	50	197	65	104	35	43	14
Graduate	213	13	202	95	185	87	165	77	158	74	207	97	202	95	131	62	95	45	123	58	144	68	127	60	103	48	128	60	64	30	33	15
Doctor	27	2	26	96	19	70	21	78	23	85	27	100	26	96	11	41	9	33	14	52	21	78	11	41	12	44	15	56	8	30	2	7
Gender																																
Female	893	56	838	94	712	80	697	78	658	74	869	97	861	96	580	65	419	47	507	57	615	69	481	54	429	48	569	64	327	37	148	17
Male	709	44	644	91	560	79	579	82	574	81	685	97	675	95	414	58	311	44	410	58	497	70	406	57	355	50	401	57	211	30	97	14
Major																																
Liberal arts	787	49	727	92	629	80	624	79	610	78	761	97	753	96	507	64	365	46	471	60	568	72	451	57	389	49	515	65	287	36	127	16
Science	363	23	343	94	283	78	290	80	270	74	358	99	349	96	223	61	162	45	191	53	234	64	191	53	171	47	205	56	105	29	58	16
Engineering	452	28	412	91	360	80	362	80	352	78	435	96	434	96	264	58	203	45	255	56	310	69	245	54	224	50	250	55	146	32	60	13

MPD, mobile phone dependence; DS, depressive symptoms.

### 3.3. Correlational analyses of variables

After controlling for participants’ grade, gender, and major, a partial correlation analysis was conducted between AP for mobile phone Internet content, RP for mobile phone Internet content, mobile phone dependence, and depressive symptoms. The results are presented in Table 2. Mobile phone dependence was significantly positively correlated with depressive symptoms (*r* = 0.488, *p* < 0.01), thus supporting *H1*. Wen et al. (56) suggested that the independent variable should be significantly correlated with the mediator variable, whereas the correlation of the moderator variable with independent and dependent variables will not have an effect on the moderating effect test. Therefore, this section will focus on explaining the correlations of AP with mobile phone dependence and depressive symptoms. The following correlation effect sizes were relatively small. AP-SRTm was significantly positively correlated with mobile phone dependence (*r* = 0.154, *p* < 0.01), while not correlated with depressive symptoms, suggesting that mobile phone dependence may be a suppressor variable. AP-NTm was significantly negatively correlated with depressive symptoms (*r* = −0.101, *p* < 0.01) but not significantly correlated with mobile phone dependence, which indicates that AP-NTm may not lead to mobile phone dependence. AP-SPTm was significantly negatively correlated with mobile phone dependence (*r* = −0.062, *p* < 0.05) and depressive symptoms (*r* = −0.091, *p* < 0.01). As the correlation effect sizes were relatively small, we further analyzed the statistical power of above correlations. The statistical power of the correlation between AP-SPTm and depressive symptoms is 0.641, and the statistical power of the correlation between AP-SPTm and mobile phone dependence is 0.489, which is less than 0.5. The statistical power is relatively weak, which suggests that the coefficient is significant due to the large sample size, and that the true correlation may be higher than 0.062. Then we explored the relationship between the two categories of the AP-SPTm, depressive symptoms and mobile phone dependence. The learning category was significantly negatively correlated with mobile phone dependence (*r* = −0.127, *p* < 0.01) and depressive symptoms (*r* = −0.128, *p* < 0.01). However, the work category was not correlated with mobile phone dependence (*r* = 0.024, *p* = 0.341) or depressive symptoms (*r* = −0.019, *p* = 0.452). This may explain the small correlation effect size between AP-SPTm, mobile phone dependence and depressive symptoms, and also indicate that work category could neither cause mobile phone dependence nor increase depressive symptoms.

**Table 2:** Correlations of mobile phone internet content preferences, mobile phone dependence, and depressive symptoms among Chinese university students (*n* = 1,602).

Variable	Mean	SD	1	2	3	4	5	6	7	8
1. DS	17.128	9.426								
2. MPD	30.914	8.934	0.488**							
3. AP-SRTm	8.447	1.349	−0.039	0.154**						
4. AP-SPTm	7.316	1.491	−0.091**	−0.062*	0.379**					
5. AP-NTm	6.710	1.828	−0.101**	−0.032	0.315**	0.442**				
6. RP-SRTm	1.263	1.762	0.272**	0.214**	0.513**	0.097**	0.062*			
7. RP-SPTm	1.313	1.696	0.069**	0.048	0.295**	0.524**	0.165**	0.318**		
8. RP-NTm	1.776	1.801	0.000	0.007	0.303**	0.287**	0.631**	0.314**	0.397**	

SD, standard deviation; MPD, mobile phone dependence; DS, depressive symptoms. *p < 0.05; **p < 0.01.

With regard to RP, RP-SRTm was significantly positively correlated with mobile phone dependence (*r* = 0.214, *p* < 0.01) and depressive symptoms (*r* = 0.272, *p* < 0.01), while RP-SPTm was significantly positively correlated with depressive symptoms (*r* = 0.069, *p* < 0.01). RP-NTm was not significantly correlated with mobile phone dependence or depressive symptoms. Based on Wen’s viewpoint (56), the correlations between RP, mobile phone dependence and depressive symptoms have limited importance in this study. Our study aims to investigate the mediating role of mobile phone dependence between different types and categories of AP and depressive symptoms, and the moderating role of different types and categories of RP between mobile phone dependence and depressive symptoms. Therefore, this study will focus on the mediating effects analysis and the moderating effects analysis.

### 3.4. Mediating effects analysis

**Figure 1:**
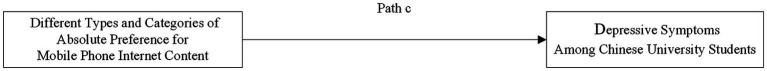
Total effect models: effect of different types and categories of absolute preference (AP) for mobile phone Internet content on depressive symptoms among Chinese university students.

**Figure 2:**
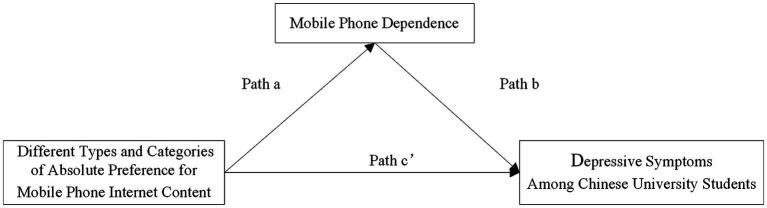
Mediation models: direct and indirect effects mediated via mobile phone dependence of absolute preference (AP) for mobile phone Internet content on depressive symptoms in Chinese university students.

Figures 1, 2 show the total effect model and the mediation model. The total effect results presented in Table 3 show that AP-SPTm and AP-NTm significantly negatively predicted depressive symptoms, whereas AP-SRTm did not. Therefore, our results support *H2b* and *H2c* but not *H2a*. The results for path a (independent → mediating) show that AP-SRTm positively predicted mobile phone dependence (*β* = 1.02, *p* < 0.001), whereas AP-SPTm negatively predicted mobile phone dependence (*β* = −0.38, *p* < 0.05). AP-NTm did not significantly predict mobile phone dependence (*β* = −0.15, *p* = 0.2078). The results of path b (mediating → dependent) show that mobile phone dependence positively predicted depressive symptoms (*β* = 0.51–0.53, all *p* < 0.001). Depressive symptoms were also negatively predicted by the lifestyle (*p* < 0.05), learning (*p* < 0.001), political news (*p* < 0.001), and social (*p* < 0.01) categories (*β* = −0.61 to −1.33).

**Table 3:** Analysis of the mediating effect of mobile phone dependence.

Effects	c	c′	a	b	ab	95% CI of ab
AP-SRTm → MPD → DS	−0.27	−0.82***	1.02***	0.53***	0.55	[0.37, 0.73]
Entertainment → MPD → DS	−0.14	−1.34***	2.24***	0.54***	1.21	[0.91,1.52]
Lifestyle → MPD → DS	−0.61*	−0.99***	0.73**	0.52***	0.38	[0.10, 0.68]
AP-SPTm → MPD → DS	−0.58***	−0.39**	−0.38*	0.51***	−0.19	[−0.35, −0.04]
Learning → MPD → DS	−1.33***	−0.70**	−1.26***	0.51***	−0.64	[−0.90, −0.37]
Work → MPD → DS	−0.18	−0.30	0.22	0.52***	0.11	[−0.13, 0.36]
AP-NTm → MPD → DS	−0.52***	−0.44***	−0.15	0.51***	−0.08	[−0.21, 0.05]
Political news → MPD → DS	−1.01***	−0.81***	−0.40	0.51***	−0.20	[−0.43, 0.02]
Social → MPD → DS	−0.70**	−0.65**	−0.10	0.51***	−0.05	[−0.29, 0.19]

c, total effect; c′, direct effect; a, effect of independent variable on mediating variable; b, effect of mediating variable on dependent variable; ab, mediating effect. MPD, mobile phone dependence; DS, depressive symptoms. *p < 0.05; **p < 0.01; ***p < 0.001.

Furthermore, we examined the mediating effect of depressive symptoms on the association between different types and categories of AP for mobile phone Internet content and depressive symptoms. First, the total effect of AP-SRTm on depressive symptoms was non-significant, while the direct and indirect effects were significant but had opposite direction of effect. These results confirm the predicted suppressing effect of mobile phone dependence, thus supporting *H3a*. For the AP-SRTm category of entertainment, the total effect on depressive symptoms was not significant, whereas the direct and indirect effects were significant but with opposite directions of effect, thus reinforcing the suppressing effect of mobile phone dependence. For the AP-SRTm category of lifestyle, although the total effect on depressive symptoms was significant, the direct and indirect effects were significant with opposite directions of effect, suggesting the suppressing effect of mobile phone dependence (57).

Second, the confidence interval of the partial mediating effect of mobile phone dependence between AP-SPTm and depressive symptoms did not contain 0, indicating statistical significance of the results and thus supporting *H3b*. Mobile phone dependence also partially mediated the effect of the AP-SPTm category of learning on depressive symptoms, but its partial mediating effect between the AP-SPTm category of work and depressive symptoms was non-significant.

Third, mobile phone dependence did not significantly mediate the effect of AP-NTm or either of its categories (i.e., political news and social) on depressive symptoms, thus supporting *H3d* but not *H3c*.

### 3.5. Moderating effects analysis

**Figure 3:**
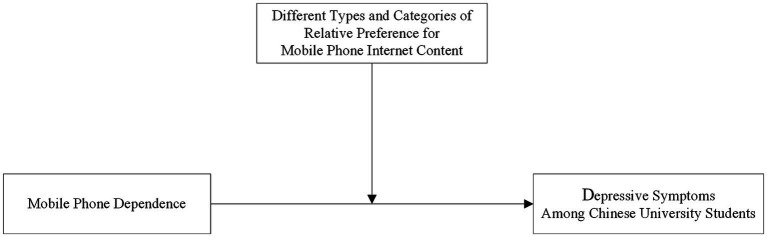
Models of the moderating effect of relative preference (RP) for mobile phone internet content on the association between mobile phone dependence and depressive symptoms.

Table 4 reports the results for tests of the moderating effects of RP for mobile phone Internet content and its categories on the association between mobile phone dependence and depressive symptoms, as modeled in Figure 3.

**Table 4:** Results for the moderating effects of relative preference (RP) for mobile phone internet content on mobile phone dependence and depressive symptoms.

Moderating variable	Variable	*β*	*t*	Significance
RP-SRTm	MPD	0.472	20.407	0.000
	RP-SRTm	0.945	8.086	0.000
	RP-SRTm*MPD	0.028	2.240	0.025
△*R*^2^ = 0.002, *F* = 5.018, *p* = 0.025				
Entertainment	MPD	0.478	20.434	0.000
	Entertainment	1.216	6.485	0.000
	Entertainment*MPD	0.034	1.716	0.086
△*R*^2^ = 0.001, *F* = 2.946, *p* = 0.086				
Lifestyle	MPD	0.496	21.629	0.000
	Lifestyle	1.252	6.430	0.000
	Lifestyle*MPD	0.034	1.608	0.108
△*R*^2^ = 0.001, *F* = 2.584, *p* = 0.108				
RP-SPTm	MPD	0.509	22.122	0.000
	RP-SPTm	0.276	2.273	0.023
	RP-SPTm*MPD	−0.040	−2.973	0.003
△*R*^2^ = 0.004, *F* = 8.839, *p* = 0.003				
Learning	MPD	0.509	22.066	0.000
	Learning	0.426	2.393	0.017
	Learning*MPD	−0.035	−1.825	0.068
△*R*^2^ = 0.002, *F* = 3.33, *p* = 0.068				
Work	MPD	0.514	22.361	0.000
	Work	0.208	0.198	0.294
	Work*MPD	−0.061	−2.788	0.005
△*R*^2^ = 0.004, *F* = 7.775, *p* = 0.005				
RP-NTm	MPD	0.509	22.085	0.000
	RP-NTm	−0.008	−0.071	0.943
	RP-NTm*MPD	−0.041	−3.177	0.002
△*R*^2^ = 0.005, *F* = 10.090, *p* = 0.002				
Political news	MPD	0.509	22.058	0.000
	Political news	0.027	0.140	0.889
	Political news*MPD	−0.065	−3.050	0.002
△*R*^2^ = 0.004, *F* = 9.304, *p* = 0.002				
Social	MPD	0.512	22.228	0.000
	Social	−0.051	−0.255	0.799
	Social*MPD	−0.051	−2.309	0.021
△*R*^2^ = 0.003, *F* = 5.332, *p* = 0.021				

MPD, mobile phone dependence; DS, depressive symptoms.

**Figure 4:**
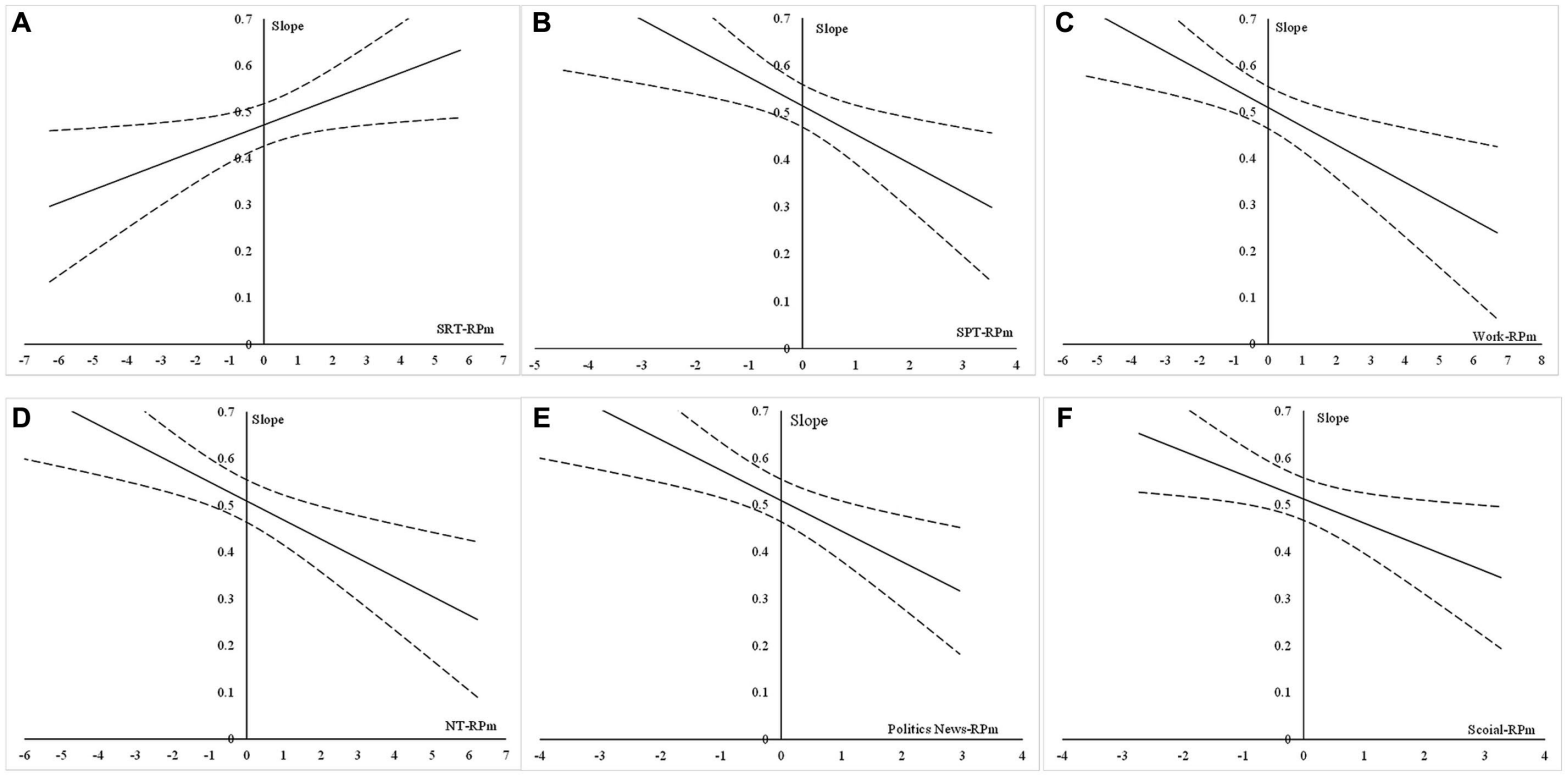
J-N plots of the moderating effects of relative preference for mobile phone Internet content (RP for mobile phone internet content). **(A)** SRT-RPm, **(B)** SPT-RPm, **(C)** Work-RPm, **(D)** NT-RPm, **(E)** Politics-RPm, and **(F)** Social-RPm.

First, RP-SRTm had a significant positive moderating effect on the association between mobile phone dependence and depressive symptoms (*β* = 0.028, *p* < 0.05). The Johnson-Neyman plot (J-N plot) in Figure 4A shows that the entire confidence band of the simple slope estimate is above 0 and that the confidence interval does not contain 0, indicating a significant moderating effect. This means that the positive effect of mobile phone dependence on depressive symptoms among Chinese university students is stronger at higher levels of RP-SRTm. Thus, *H4a* was supported. However, neither category of RP-SRTm (i.e., entertainment and lifestyle) was found to significantly moderate the mobile phone dependence–depressive symptoms association.

Second, RP-SPTm had a significant negative moderating effect on the association between mobile phone dependence and depressive symptoms (*β* = −0.040, *p* < 0.01). The J-N plot in Figure 4B shows that the entire confidence band of the simple slope estimate is above 0 and that the confidence interval does not contain 0, indicating a significant moderating effect. Thus, *H4b* was supported. The RP-SPTm category of work also had a significant moderating effect on the mobile phone dependence–depressive symptoms association (*β* = −0.061, *p* < 0.01). In the J-N plot of Figure 4C, the entire confidence band of the simple slope estimate is above 0 and the confidence interval does not contain 0, indicating a significant moderating effect. These results indicate that the positive effect of mobile phone dependence on depressive symptoms among Chinese university students is weaker at higher levels of RP-SPTm and its work category. However, the RP-SPTm category of learning did not have a significant moderating effect on the mobile phone dependence–depressive symptoms association.

Third, RP-NTm had a significant negative moderating effect on the association between mobile phone dependence and depressive symptoms (*β* = −0.041, *p* < 0.01). The J-N plot in Figure 4D shows that the entire confidence band of the simple slope estimate is above 0 and that the confidence interval does not contain 0, indicating a significant moderating effect. Thus, *H4c* was supported. The RP-NTm category of political news also had a significant negative moderating effect on the mobile phone dependence–depressive symptoms association (*β* = −0.065, *p* < 0.01). In the J-N plot in Figure 4E, the entire confidence band of the simple slope estimate is above 0 and the confidence interval does not contain 0, indicating a significant moderating effect. Finally, the RP-NTm category social category also had a significant negative moderating effect on the association between mobile phone dependence and depressive symptoms (*β* = −0.051, *p* < 0.05). The J-N plot in Figure 4F shows that the entire confidence band of the simple slope estimate is above 0 and that the confidence interval does not contain 0, indicating a significant moderating effect. These results indicate that the positive effect of mobile phone dependence on depressive symptoms among Chinese university students is weaker at higher levels of RP-NTm and its political news and social categories.

The horizontal axis represents RP for mobile phone Internet content, the vertical axis represents the slope of mobile phone dependence on depressive symptoms among Chinese university students, and the dashed lines represent the 95% confidence interval.

## 4. Discussion

Using a sample of Chinese university students, this study explored associations among mobile phone dependence, depressive symptoms, and AP and RP for mobile phone Internet content. As predicted, we found a positive association between mobile phone dependence and depressive symptoms. The mediating role of mobile phone dependence between AP for mobile phone Internet content and depressive symptoms differed according to the type of information content. Moreover, RP for mobile phone Internet content with different types of information content moderated the association between mobile phone dependence and depressive symptoms in different directions.

### 4.1. Mediating influence of mobile phone dependence

The mediation analysis showed that the total effect of AP-SRTm on students’ depressive symptoms was non-significant, indicating that mobile phone dependence suppressed the association. By contrast, AP-SPTm was a significant negative predictor of students’ depressive symptoms, with mobile phone dependence partially mediating this association. Although AP-NTm directly negatively predicted students’ depressive symptoms, this association was not mediated by mobile phone dependence.

Similarly, previous studies have found a positive association between mobile phone dependence and depressive symptoms in Chinese university students (6, 17, 18). With regard to the suppressing effect of mobile phone dependence, the direct and indirect effects of AP-SRTm on Chinese university students’ depressive symptoms had opposite directions. Without controlling for the mediation effects of mobile phone dependence, the total effect of AP-SRTm on depressive symptoms was non-significant; however, after controlling for mobile phone dependence, AP-SRTm significantly negatively predicted depressive symptoms (*β* = −0.817, *p* < 0.001), indicating that mobile phone dependence had a complete suppressing effect. This suppressing effect is a generalized mediation effect and clearly explains why the total effect of AP-SRTm on depressive symptoms was non-significant—the direct effect was suppressed by mobile phone dependence.

Our findings differ from the conventional view. There are several plausible explanations. First, the results may be influenced by different research periods, contexts, and populations, especially regarding the type and extent of negative effects (58). Second, the descriptive statistics showed that over 95% of Chinese university students in our final sample had AP-SRTm (i.e., entertainment and lifestyle information), which is much higher than their AP for other types. This partly reflects the prevalence of AP-SRTm in our focal population in the post-pandemic era. It is also possible that, compared with other types, AP-SRTm has become a basic need for Chinese university students. Similarly, scholars have found that, during the pandemic, Chinese university students preferred going online to play games, watch videos, read novels, or access other stress-reducing Internet content in their spare time (59). In the short term, this online behavior becomes an inherent behavior pattern with certain stability and brings a sense of pleasure to Chinese university students (59). However, in the long term, maintaining such behavior ceases to provide the stimulus to curb depressive symptoms, instead increasing mobile phone dependence and, in turn, depressive symptoms in Chinese university students. Finally, the compensatory Internet use theory (60) provides a complementary explanation for how AP-SRTm can lead to mobile phone dependence and increase depressive symptoms. This theory emphasizes that, although people go online to escape real-life problems or relieve irritability, these efforts to mitigate negative emotions may lead to negative consequences, such as addiction. In the context of our study, Chinese university students in the post-pandemic era may have developed AP-SRTm to relieve irritability, increasing their tendency toward mobile phone dependence and reinforcing their depressive symptoms.

Some scholars suggest that meeting autonomous needs positively affects personal well-being (61). In particular, when individuals are free to choose behaviors that satisfy autonomous needs, individual self-esteem and self-confidence are enhanced, thereby boosting individual motivation and improving emotional states (62). Therefore, after controlling for mobile phone dependence, when Chinese university students freely choose any type of mobile phone Internet content to satisfy their autonomous needs, their mood and depressive symptoms can improve.

However, according to the flow theory, a match between individual abilities and task challenges leads to a flow state, an experience characterized by loss of self-awareness and a sense of time distortion. Flow can result in the formation of mobile phone dependence. However, without the match, they will experience boredom, apathy, and anxiety. Studies have also shown that the individual abilities required to search for, read, and use stress-reducing mobile phone Internet content (i.e., entertainment and lifestyle information) are more easily matched with task challenges. As individuals concentrate on these tasks, they ignore changes in the external environment, lose track of time, and become determined to invest even more time (63). By devoting increasing attention to these information categories, their desire to keep using them grows stronger and they become addicted rapidly (64); the mobile phone dependence thus reinforces depressive symptoms. By contrast, the challenging tasks characteristic of stress-producing Internet content seldom match individual abilities. Consequently, this type of information can easily lead to psychological tension and negative emotional experiences, as users cannot obtain continuous positive feedback. Without losing track of time, it is also quite difficult for users to immerse themselves in stress-producing content, such as learning information, which interrupts their efforts to learn. As a result, stress-producing mobile phone Internet content curbs the formation of mobile phone dependence and reduces depressive symptoms.

Therefore, to prevent and mitigate Chinese university students’ mobile phone dependence and depressive symptoms in the post-pandemic era, China should focus on guiding students to form better AP for mobile phone Internet content. Neither stress-producing mobile phone Internet content (e.g., work information) nor neutral mobile phone Internet content (i.e., political news and social information) generates mobile phone dependence. However, mobile phone Internet content, except for the work category, can partly curb depressive symptoms. At the same time, it is necessary to dialectically view the stress-reducing mobile phone Internet content_−_ (i.e., entertainment and lifestyle information), which can lead to mobile phone dependence but also reduce depressive symptoms.

### 4.2. Moderating influence of RP for mobile phone internet content

Our results showed that, in the post-pandemic era, different types of RP for mobile phone Internet content moderated the mobile phone dependence–depressive symptoms association in opposite directions: RP-SRTm had a significant positive moderating effect, whereas RP-SPTm and RP-NTm had significant negative moderating effects.

Studies have shown that the level of social distance significantly affects the intensity of the third-person effect for Internet information, with intensity increasing as the social distance increases between the audience and others (42). Higher RP for mobile phone Internet content indicates a larger difference between the respective frequencies of mobile phone Internet exposure to and offline communication regarding the same type of information. Detachment from others in daily life increases social distance, thus intensifying the third-person effect and moderating the effect of mobile phone dependence on depressive symptoms. In the post-pandemic era, Chinese university students’ access to the stress-producing type (i.e., learning and work information) and the neutral type (i.e., political news and social information) of mobile phone Internet content can easily trigger negative emotions. As people with a high (vs. low) RP-SPTm and RP-NTm are more likely to obtain the stress-producing and neutral types of information online, they do not frequently communicate offline regarding either type of information. Consequently, their level of negative emotion is lower and their depressive symptoms are milder. Drawing on the third-person effect theory with respect to information eliciting negative emotions (41), RP-SPTm and RP-NTm can negatively moderate the effect of mobile phone dependence on depressive symptoms, thus reducing depressive symptoms in Chinese university students.

In addition, students’ access to stress-reducing mobile phone Internet content (i.e., entertainment and lifestyle information) can generate pleasure and positive emotions, while the third-person effect theory proposes that people tend to overestimate the effect of stress-reducing mobile phone Internet content. As we proposed, although stress-reducing mobile phone Internet content can produce short-term flow experiences and increase positive emotions, prolonged exposure to stress-reducing mobile phone Internet content can result in more negative emotions. Since our investigation began in February 2023, the findings were more focused on the long-term effects in the post-pandemic era, while the negative emotions were considered to be amplified by the third-person effect. Moreover, RP for mobile phone Internet content increased the social distance and intensified the third-person effect, indicating the positive moderating effect of RP-SRTm. Furthermore, Chinese culture emphasizes that “shared joy with others is more enjoyable,” which may indicate that exchanging stress-reducing information is a pleasure. People with a high RP-SRTm do not frequently exchange this type of information with others offline. Therefore, university students with a high (vs. low) RP-SRTm may have less overall access to the stress-reducing type of information, lower levels of positive emotions, and perhaps more severe depressive symptoms. Based on this, RP-SRTm can positively moderate the effect of mobile phone dependence on depressive symptoms in Chinese university students, resulting in higher depressive symptoms.

The aforementioned analysis indicates the need for Chinese university students to engage strategically in offline communication with others in daily life, based on different categories and types of information. In particular, greater offline communication regarding stress-reducing information (i.e., entertainment and lifestyle) can reduce RP-SRTm, while less communication about stress-producing information (i.e., learning and work) and neutral information (i.e., political news and social) can increase RP-SPTm and RP-NTm. Such strategic communication can serve as a “psychological vaccine” to prevent mobile phone dependence and reduce depressive symptoms in Chinese university students.

## 5. Limitations and future research

This study had several limitations. First, given its cross-sectional design, the study could not detect causality. This should be addressed in the future through longitudinal studies. Second, as the sample included only Chinese university students, the findings have low external validity and further research should explore their generalizability to other populations or to university students in other countries. Third, we used a tool to evaluate depressive symptoms rather than a diagnostic assessment of depression, so any conclusions cannot be generalized to the clinical population. Finally, though this study explored the effects of different types and categories of APs on mobile phone dependence and depressive symptoms, there were cases where relatively small effect sizes exist between certain categories of APs, mobile phone dependence, and depressive symptoms. The reasons are as follows. (1) Smaller statistical power indicates that the significance is supported by the large sample size, and therefore the true correlation is higher than the statistical value. (2) There may be a situation where a certain category of a certain type of AP (e.g., the work category of AP-SPTm) is less or insignificantly correlated with mobile phone dependence or depressive symptoms, thus reducing the value of the correlation for that type of AP. (3) There may be factors other than AP that contribute to mobile phone dependence. Therefore, we suggest that future research could further elaborate the types, categories, and measurement of preferences for mobile phone Internet content, as well as supplement other factors that could contribute to mobile phone dependence.

## 6. Conclusion

Our study produced three main findings. First, mobile phone dependence is positively associated with depressive symptoms among Chinese university students in the post-pandemic era. Second, mobile phone dependence suppressed the effect of AP-SRTm on depressive symptoms. One plausible explanation is that AP-SRTm has become a basic need for Chinese university students to access the Internet, while mobile phone dependence could mediate the effect of AP-SPTm on depressive symptoms among Chinese university students. Third, RP-SRTm had a significant positive moderating effect on the association between mobile phone dependence and depressive symptoms, whereas RP-SPTm and RP-NTm had a significant negative moderating effect on the aforementioned association. Our study extends the understanding of AP and RP for mobile phone Internet content, mobile phone dependence, and depressive symptoms among Chinese university students. The insights provided by our results could help students to adjust their AP and RP for Internet content, thereby lowering their depressive symptoms.

## Data availability statement

The original contributions presented in the study are included in the article/supplementary material, further inquiries can be directed to the corresponding author.

## Ethics statement

The studies involving humans were approved by Affiliated Chongqing University Cancer Hospital Ethics Committee. The studies were conducted in accordance with the local legislation and institutional requirements. The participants provided their written informed consent to participate in this study. Written informed consent was obtained from the individual(s) for the publication of any potentially identifiable images or data included in this article.

## Author contributions

HY and YJ conceived the idea of this study and collected the necessary data. HY, YJ, ZW, and JT wrote the original draft of this manuscript. All authors contributed to the article and approved the submitted version.

## Abbreviations

AP: absolute preference
AP-SRTm: absolute preference for the stress-reducing type of mobile phone internet content
AP-SPTm: absolute preference for the stress-producing type of mobile phone internet content
AP-NTm: absolute preference for the neutral type of mobile phone internet content
RP: relative preference
RP-SRTm: relative preference for the stress-reducing type of mobile phone internet content
RP-SPTm: relative preference for the stress-producing type of mobile phone internet content
RP-NTm: relative preference for the neutral type of mobile phone internet content
